# Supplementary material for: The bZIP Transcription Factors in Current Jasmine Genomes: Identification, Characterization, Evolution and Expressions
Source: Int J Mol Sci. 2023 Dec 29;25(1):488. doi: 10.3390/ijms25010488 (PMC10779407; doi:10.3390/ijms25010488)
Supplement: Supplementary file 1 [file ijms-25-00488-s001.zip › ijms-2734841-supplementary.pdf]

Supplementary Material

Table S1a Physicochemical properties of *SJbZIP* genes

Gene name	Gene ID	Number of Amino Acid	Molecular Weight	Theoretical pI	Instability Index	Aliphatic Index	Grand Average of Hydropathicity
SJbZIP01	SJ00480.t1	359	39006.78	5.62	57.91	61.23	-0.719
SJbZIP02	SJ01012.t1	329	36806.44	8.96	61.15	82.28	-0.525
SJbZIP03	SJ04867.t1	461	51854.67	8.66	61.57	85.51	-0.454
SJbZIP04	SJ05313.t1	137	15580.78	6.29	51.89	99.56	-0.511
SJbZIP05	SJ06608.t1	440	49042.94	7.83	52.92	67.98	-0.704
SJbZIP06	SJ06896.t1	324	35824.41	9.44	52.41	73.15	-0.803
SJbZIP07	SJ07080.t1	139	16877.6	9.03	71.27	84.17	-0.692
SJbZIP08	SJ07347.t1	195	22695.81	10.55	68.92	73.9	-0.878
SJbZIP09	SJ07599.t1	778	84095.11	5.67	48.16	70.03	-0.572
SJbZIP10	SJ08602.t1	398	44326.66	9.56	56.2	62.41	-0.953
SJbZIP11	SJ09847.t1	170	19731.3	4.82	58.71	66.53	-0.746
SJbZIP12	SJ10412.t1	324	36137.83	9.32	63.81	79.94	-0.549
SJbZIP13	SJ11252.t1	283	32167.74	6.38	70.71	71.34	-0.912
SJbZIP14	SJ11562.t1	282	31810.25	8.56	54.18	78.12	-0.734
SJbZIP15	SJ11566.t1	251	27718.38	5.81	51.28	76.14	-0.659
SJbZIP16	SJ11825.t1	159	17665.82	7.75	62.53	69.31	-0.604
SJbZIP17	SJ12250.t1	249	27527.77	6.51	45.82	68.55	-0.802
SJbZIP18	SJ12717.t1	424	45571.2	9.81	58.69	60.5	-0.692
SJbZIP19	SJ12860.t1	160	17795.7	5.61	49.16	74.44	-0.672
SJbZIP20	SJ12999.t1	278	29892.21	6.24	43.57	67.12	-0.664
SJbZIP21	SJ13279.t1	447	48173.08	5.85	62.77	63.31	-0.775
SJbZIP22	SJ14539.t1	556	60602.74	7.06	68.01	59.35	-0.883
SJbZIP23	SJ14953.t1	382	41603.33	9.11	55.86	61.31	-0.912
SJbZIP24	SJ15190.t1	360	41301.95	8.55	49.69	76.17	-0.58
SJbZIP25	SJ15219.t1	468	51848.34	6.8	47.92	63.8	-0.867
SJbZIP26	SJ15245.t1	138	15623.62	7.79	56.53	63.04	-0.77
SJbZIP27	SJ15499.t1	306	34170.68	4.81	60.49	90.78	-0.475
SJbZIP28	SJ15548.t1	306	34158.58	4.77	59.77	89.84	-0.495
SJbZIP29	SJ15769.t1	370	40590.67	9.44	52.78	65.59	-0.792
SJbZIP30	SJ16381.t1	347	36415.72	5.48	55.7	45.94	-0.859
SJbZIP31	SJ16982.t1	174	20602.93	5.39	56.68	67.18	-1.013
SJbZIP32	SJ17391.t1	112	13326.29	10.12	84.79	80.09	-1.129
SJbZIP33	SJ17779.t1	409	44846.56	6.01	50.5	62.57	-0.886
SJbZIP34	SJ18037.t1	137	16007.29	9.07	71.31	78.98	-0.7
SJbZIP35	SJ18569.t1	174	20591.91	5.82	60.4	76.21	-0.972
SJbZIP36	SJ19200.t1	447	47880.35	9.39	51.53	67.23	-0.655
SJbZIP37	SJ19414.t1	168	19046.24	6.41	58.49	63.99	-0.775
SJbZIP38	SJ19457.t1	453	50020.34	8.83	62.3	71.02	-0.687

SJbZIP39	SJ19688.t1	369	41449.19	6.68	50.64	88.35	-0.303
SJbZIP40	SJ22524.t1	329	36514.52	8	57.4	58.54	-0.91
SJbZIP41	SJ22530.t1	302	33315.79	7.73	47.75	76.23	-0.664
SJbZIP42	SJ22657.t1	132	15617.02	9.92	65.31	80.53	-0.758
SJbZIP43	SJ22770.t1	151	16526.42	9.74	74.21	66.62	-1.034
SJbZIP44	SJ23101.t1	395	42389.72	7.08	60.1	48.03	-0.943
SJbZIP45	SJ23326.t1	297	32814.07	4.99	44.92	62.66	-0.641
SJbZIP46	SJ24332.t1	457	49833.69	6.54	56.84	76.26	-0.477
SJbZIP47	SJ24475.t1	201	23333.91	5.08	59.51	72.34	-0.812
SJbZIP48	SJ25515.t1	264	29726.52	9.16	49.83	73.22	-0.775
SJbZIP49	SJ25666.t1	426	47000.21	7.84	51.55	83.26	-0.339
SJbZIP50	SJ26323.t1	462	49325.26	5.9	56.44	57.25	-0.764
SJbZIP51	SJ26606.t1	302	32200.05	5.43	56.82	66.13	-0.535
SJbZIP52	SJ26761.t1	411	44294.61	9.74	49.81	59.78	-0.7
SJbZIP53	SJ26833.t1	152	17474.38	5.9	64.19	77.17	-0.874
SJbZIP54	SJ26872.t1	455	50582.88	5.93	59.65	65.63	-0.866
SJbZIP55	SJ26892.t1	252	27725.03	6.39	40.49	69.29	-0.701
SJbZIP56	SJ28781.t1	305	34143.12	5.96	65.36	60.2	-0.861
SJbZIP57	SJ29591.t1	144	16394.59	6.59	53.34	81.32	-0.622
SJbZIP58	SJ29971.t1	164	19065.34	5.3	52.62	82.62	-0.73
SJbZIP59	SJ29974.t1	164	19237.53	5.05	52.53	81.46	-0.729
SJbZIP60	SJ32383.t1	294	33095.27	9.04	54.1	66.02	-0.832
SJbZIP61	SJ32459.t1	412	46402.85	6.57	54.04	74.17	-0.723
SJbZIP62	SJ32583.t1	506	56290.95	6.5	64.57	73.6	-0.607
SJbZIP63	SJ32909.t1	490	54228.22	8.65	64.76	65.76	-0.632
SJbZIP64	SJ32921.t1	405	44740.86	8.77	53.5	60.4	-0.853

Table S1b Physicochemical properties of *DJbZIP* genes

Gene name	Gene ID	Number of Amino Acid	Molecular Weight	Theoretical pI	Instability Index	Aliphatic Index	Grand Average of Hydropathicity
DJbZIP01	DJ00457.t1	359	39012.8	5.62	57.33	61.23	-0.707
DJbZIP02	DJ00925.t1	329	36797.43	8.96	62.55	82.28	-0.526
DJbZIP03	DJ01555.t1	403	42638.33	5.68	62.42	42.01	-0.968
DJbZIP04	DJ04535.t1	482	53713.54	6.65	62.05	86.47	-0.403
DJbZIP05	DJ04965.t1	137	15580.78	6.29	51.89	99.56	-0.511
DJbZIP06	DJ06237.t1	483	53949.47	8.35	57.41	68.8	-0.698
DJbZIP07	DJ06524.t1	324	35824.41	9.44	52.41	73.15	-0.803
DJbZIP08	DJ06709.t1	139	16877.6	9.03	71.27	84.17	-0.692
DJbZIP09	DJ06993.t1	195	22695.81	10.55	68.92	73.9	-0.878
DJbZIP10	DJ07226.t1	778	84108.06	5.6	48.07	69.64	-0.575
DJbZIP11	DJ08186.t1	399	44277.65	9.53	54.54	62.76	-0.929

DJbZIP12	DJ09450.t1	170	19731.3	4.82	58.71	66.53	-0.746
DJbZIP13	DJ09971.t1	324	36137.83	9.32	63.81	79.94	-0.549
DJbZIP14	DJ10813.t1	283	32139.68	6.38	69.16	70.64	-0.921
DJbZIP15	DJ11122.t1	282	31810.25	8.56	54.18	78.12	-0.734
DJbZIP16	DJ11126.t1	251	27718.38	5.81	51.28	76.14	-0.659
DJbZIP17	DJ11407.t1	159	17665.82	7.75	62.53	69.31	-0.604
DJbZIP18	DJ11800.t1	249	27527.77	6.51	45.82	68.55	-0.802
DJbZIP19	DJ12235.t1	424	45571.2	9.81	58.69	60.5	-0.692
DJbZIP20	DJ12377.t1	160	17795.7	5.61	49.16	74.44	-0.672
DJbZIP21	DJ12500.t1	278	29908.21	6.24	42.82	66.76	-0.673
DJbZIP22	DJ12766.t1	447	48173.08	5.85	62.77	63.31	-0.775
DJbZIP23	DJ14042.t1	556	60602.74	7.06	68.01	59.35	-0.883
DJbZIP24	DJ14446.t1	382	41603.33	9.11	55.86	61.31	-0.912
DJbZIP25	DJ14967.t1	174	20589.93	5.39	56.68	67.18	-0.997
DJbZIP26	DJ15494.t1	439	46715.91	6.18	51.53	56.54	-0.684
DJbZIP27	DJ16083.t1	397	43609.24	9.16	54.84	71.96	-0.669
DJbZIP28	DJ16287.t1	306	34158.58	4.77	59.77	89.84	-0.495
DJbZIP29	DJ16543.t1	138	15637.64	7.79	57.15	63.77	-0.754
DJbZIP30	DJ16564.t1	468	51749.21	6.64	47.95	63.8	-0.858
DJbZIP31	DJ16591.t1	360	41273.89	8.55	49.93	75.64	-0.586
DJbZIP32	DJ17098.t1	510	56212.46	6.02	47.4	67.73	-0.744
DJbZIP33	DJ17368.t1	137	15948.22	8.8	73.58	78.98	-0.679
DJbZIP34	DJ17884.t1	174	20591.91	5.82	60.4	76.21	-0.972
DJbZIP35	DJ18514.t1	447	47880.35	9.39	51.53	67.23	-0.655
DJbZIP36	DJ18706.t1	168	19046.24	6.41	58.49	63.99	-0.775
DJbZIP37	DJ18752.t1	453	50102.43	9.07	61.31	71.66	-0.703
DJbZIP38	DJ18962.t1	369	41479.28	6.67	48.59	88.62	-0.288
DJbZIP39	DJ21442.t1	329	36474.45	8	56.19	58.84	-0.899
DJbZIP40	DJ21448.t1	302	33331.79	7.73	48.38	75.89	-0.672
DJbZIP41	DJ21566.t1	132	15615.05	9.83	57.41	83.48	-0.714
DJbZIP42	DJ21683.t1	151	16526.42	9.74	74.21	66.62	-1.034
DJbZIP43	DJ21994.t1	395	42403.74	7.08	60.41	48.28	-0.937
DJbZIP44	DJ22225.t1	297	32819.06	4.92	46.79	61.35	-0.639
DJbZIP45	DJ23211.t1	458	49920.77	6.54	57.07	76.09	-0.477
DJbZIP46	DJ23345.t1	201	23333.91	5.08	59.51	72.34	-0.812
DJbZIP47	DJ24288.t1	264	29726.52	9.16	49.83	73.22	-0.775
DJbZIP48	DJ24433.t1	362	39854.14	8.96	50	82.9	-0.331
DJbZIP49	DJ25082.t1	462	49250.13	5.74	56.56	57.25	-0.765
DJbZIP50	DJ25331.t1	300	32044.9	5.43	57.77	66.57	-0.527
DJbZIP51	DJ25493.t1	411	44324.63	9.66	49.55	59.78	-0.691
DJbZIP52	DJ25568.t1	152	17474.38	5.9	64.19	77.17	-0.874
DJbZIP53	DJ25605.t1	455	50620.89	6.14	60.19	64.77	-0.885
DJbZIP54	DJ25624.t1	252	27725.03	6.39	40.49	69.29	-0.701
DJbZIP55	DJ27417.t1	305	34215.22	6.05	64.56	60.82	-0.877

DJbZIP56	DJ29167.t1	164	19237.53	5.05	52.53	81.46	-0.729
DJbZIP57	DJ29170.t1	164	19065.34	5.3	52.62	82.62	-0.73
DJbZIP58	DJ29537.t1	144	16380.56	6.59	53.34	81.32	-0.623
DJbZIP59	DJ30851.t1	294	33095.27	9.04	54.1	66.02	-0.832
DJbZIP60	DJ30931.t1	412	46362.78	6.57	53.92	74.42	-0.715
DJbZIP61	DJ31038.t1	505	56221.84	6.5	64.29	73.15	-0.621
DJbZIP62	DJ31358.t1	490	54225.31	8.77	63.3	65.96	-0.629
DJbZIP63	DJ31370.t1	413	45654.99	8.57	54.19	63	-0.802

Table S1c Physicochemical properties of *HTbZIP* genes

Gene name	Gene ID	Number of Amino Acid	Molecular Weight	Theoretical pI	Instability Index	Aliphatic Index	Grand Average of Hydropathicity
HTbZIP01	HT00474.t1	359	39006.78	5.62	57.91	61.23	-0.719
HTbZIP02	HT00942.t1	371	41648.93	6.68	67.21	83.45	-0.508
HTbZIP03	HT01556.t1	403	42557.23	5.57	60.86	41.54	-0.957
HTbZIP04	HT04590.t1	482	53817.61	6.54	61.88	85.66	-0.425
HTbZIP05	HT05041.t1	137	15580.78	6.29	51.89	99.56	-0.511
HTbZIP06	HT06333.t1	483	53949.47	8.35	57.41	68.8	-0.698
HTbZIP07	HT06610.t1	324	35824.41	9.44	52.41	73.15	-0.803
HTbZIP08	HT06803.t1	139	16861.6	9.19	73.61	81.37	-0.734
HTbZIP09	HT07070.t1	195	22695.81	10.55	68.92	73.9	-0.878
HTbZIP10	HT07296.t1	778	84108.06	5.6	48.07	69.64	-0.575
HTbZIP11	HT08265.t1	397	44245.6	9.56	56.27	62.32	-0.945
HTbZIP12	HT09538.t1	169	19708.22	4.73	58.34	66.86	-0.754
HTbZIP13	HT10088.t1	448	49970.46	9.89	59.26	92.41	-0.236
HTbZIP14	HT10882.t1	283	32169.71	6.38	71.39	70.64	-0.923
HTbZIP15	HT11194.t1	282	31810.25	8.56	54.18	78.12	-0.734
HTbZIP16	HT11199.t1	251	27718.38	5.81	51.28	76.14	-0.659
HTbZIP17	HT11475.t1	159	17665.82	7.75	62.53	69.31	-0.604
HTbZIP18	HT12259.t1	424	45585.22	9.81	58.24	60.73	-0.693
HTbZIP19	HT12403.t1	160	17795.7	5.61	49.16	74.44	-0.672
HTbZIP20	HT12522.t1	278	29869.19	6.07	41.51	67.12	-0.64
HTbZIP21	HT12766.t1	447	48173.08	5.85	62.77	63.31	-0.775
HTbZIP22	HT14037.t1	556	60602.74	7.06	68.01	59.35	-0.883
HTbZIP23	HT14452.t1	382	41547.22	9.11	54.09	60.29	-0.925
HTbZIP24	HT14684.t1	347	39767.19	7.74	48.79	77.64	-0.573
HTbZIP25	HT14711.t1	468	51848.34	6.8	47.92	63.8	-0.867
HTbZIP26	HT14729.t1	138	15623.62	7.79	56.53	63.04	-0.77
HTbZIP27	HT14993.t1	306	34158.58	4.77	59.77	89.84	-0.495
HTbZIP28	HT15192.t1	372	40718.8	9.44	53.65	65.51	-0.789
HTbZIP29	HT15805.t1	407	43288.85	5.65	52.88	54.28	-0.756
HTbZIP30	HT16365.t1	174	20602.93	5.39	56.68	67.18	-1.013

HTbZIP31	HT16779.t1	112	13326.29	10.12	84.79	80.09	-1.129
HTbZIP32	HT17169.t1	409	44891.64	5.91	50.77	63.03	-0.876
HTbZIP33	HT17432.t1	137	15991.25	9.07	74.13	76.13	-0.739
HTbZIP34	HT18007.t1	174	20578.91	5.83	62	76.21	-0.957
HTbZIP35	HT18661.t1	451	48010.45	9.3	52.47	66.85	-0.646
HTbZIP36	HT18874.t1	168	19046.24	6.41	58.49	63.99	-0.775
HTbZIP37	HT18919.t1	453	50102.43	9.07	61.73	71.43	-0.702
HTbZIP38	HT19139.t1	369	41449.19	6.68	50.64	88.35	-0.303
HTbZIP39	HT21682.t1	329	36514.52	8	57.4	58.54	-0.91
HTbZIP40	HT21688.t1	302	33331.79	7.73	48.38	75.89	-0.672
HTbZIP41	HT21813.t1	132	15613.08	9.87	60.33	83.48	-0.683
HTbZIP42	HT21923.t1	151	16526.42	9.74	74.21	66.62	-1.034
HTbZIP43	HT22222.t1	461	49974.55	7.76	59.13	57.2	-0.791
HTbZIP44	HT22447.t1	297	32859.07	4.87	46.14	62.66	-0.641
HTbZIP45	HT23451.t1	458	49919.74	6.54	57.8	76.09	-0.484
HTbZIP46	HT23585.t1	201	23333.91	5.08	59.51	72.34	-0.812
HTbZIP47	HT24525.t1	264	29726.52	9.16	49.83	73.22	-0.775
HTbZIP48	HT24674.t1	455	50287.79	7	50.4	82	-0.339
HTbZIP49	HT25341.t1	462	49209.14	5.61	56.56	58.1	-0.739
HTbZIP50	HT25619.t1	340	36601.14	7.7	53.63	66.47	-0.554
HTbZIP51	HT25791.t1	411	44213.43	9.83	48.89	58.83	-0.711
HTbZIP52	HT25864.t1	152	17474.38	5.9	64.19	77.17	-0.874
HTbZIP53	HT25900.t1	455	50599.87	6.08	59.81	64.77	-0.875
HTbZIP54	HT25922.t1	252	27725.03	6.39	40.49	69.29	-0.701
HTbZIP55	HT27822.t1	305	34287.28	5.86	63.29	60.82	-0.867
HTbZIP56	HT28606.t1	144	16394.59	6.59	53.34	81.32	-0.622
HTbZIP57	HT28998.t1	110	12754.22	6.1	46.28	69.18	-0.784
HTbZIP58	HT29008.t1	164	19051.31	5.3	52.62	82.01	-0.732
HTbZIP59	HT29012.t1	164	19237.53	5.05	52.53	81.46	-0.729
HTbZIP60	HT31417.t1	294	33065.18	9.04	54.1	66.02	-0.841
HTbZIP61	HT31601.t1	537	59752.88	6.5	64.03	72.96	-0.575
HTbZIP62	HT31921.t1	471	52047.74	8.6	62.56	66.14	-0.63
HTbZIP63	HT31933.t1	405	44854.02	8.77	53.24	60.64	-0.848

Table S2 Codon bias parameters of *HTbZIP* genes

ID	U3s	C3s	A3s	G3s	CAI	CBI	Fop	ENC	GC3s	GC	GC1	GC2	GC12
HTbZIP01	0.4468	0.2057	0.2939	0.3011	0.191	-0.071	0.398	57.29	0.401	0.479	0.561	0.456	0.508
HTbZIP02	0.4498	0.2042	0.3034	0.2836	0.176	-0.062	0.383	51.64	0.386	0.465	0.557	0.422	0.489
HTbZIP03	0.4725	0.178	0.3488	0.2268	0.202	-0.065	0.41	51.01	0.327	0.489	0.567	0.542	0.554
HTbZIP04	0.4078	0.2078	0.4016	0.2219	0.161	-0.165	0.325	55.5	0.338	0.448	0.561	0.422	0.492
HTbZIP05	0.4057	0.2453	0.2545	0.3431	0.205	0.038	0.417	55.08	0.462	0.474	0.573	0.370	0.471
HTbZIP06	0.3967	0.2397	0.3333	0.2786	0.186	-0.091	0.379	57.14	0.41	0.469	0.519	0.444	0.481
HTbZIP07	0.3237	0.2116	0.2894	0.3985	0.145	-0.145	0.33	55.17	0.497	0.51	0.563	0.452	0.508
HTbZIP08	0.3474	0.2842	0.4	0.3295	0.193	0	0.419	50.43	0.434	0.4	0.421	0.307	0.364
HTbZIP09	0.3133	0.2467	0.3709	0.3286	0.149	-0.041	0.387	52.64	0.446	0.448	0.454	0.413	0.434
HTbZIP10	0.438	0.2088	0.276	0.3214	0.194	-0.083	0.371	53.17	0.415	0.466	0.528	0.437	0.482
HTbZIP11	0.3379	0.215	0.3765	0.3312	0.173	-0.094	0.37	55.73	0.427	0.475	0.553	0.427	0.490
HTbZIP12	0.4833	0.15	0.3645	0.3737	0.174	-0.1	0.375	48.49	0.362	0.383	0.424	0.300	0.362
HTbZIP13	0.4073	0.2191	0.3426	0.2448	0.185	0.009	0.417	56.14	0.375	0.458	0.532	0.437	0.484
HTbZIP14	0.4227	0.2045	0.3868	0.28	0.17	-0.128	0.348	48.47	0.366	0.431	0.525	0.387	0.456
HTbZIP15	0.4306	0.1818	0.2611	0.3721	0.151	-0.1	0.348	53.79	0.442	0.475	0.544	0.410	0.477
HTbZIP16	0.4385	0.1711	0.2613	0.3797	0.172	-0.107	0.354	53.85	0.435	0.478	0.556	0.413	0.484
HTbZIP17	0.4526	0.2774	0.2051	0.2793	0.208	0.027	0.432	52.81	0.445	0.47	0.469	0.475	0.472
HTbZIP18	0.3739	0.1216	0.389	0.3155	0.144	-0.119	0.354	48.51	0.361	0.469	0.515	0.499	0.507
HTbZIP19	0.3952	0.2661	0.1864	0.4248	0.183	-0.071	0.388	52.12	0.533	0.485	0.509	0.385	0.447
HTbZIP20	0.5087	0.2174	0.3333	0.2	0.209	-0.086	0.383	51.03	0.325	0.453	0.559	0.466	0.513
HTbZIP21	0.384	0.2092	0.2893	0.3507	0.21	-0.014	0.422	53.84	0.45	0.503	0.574	0.462	0.518
HTbZIP22	0.4473	0.1733	0.3356	0.2955	0.165	-0.178	0.33	54.15	0.369	0.454	0.517	0.454	0.486
HTbZIP23	0.4227	0.2096	0.3529	0.2575	0.198	-0.032	0.412	53.07	0.374	0.459	0.525	0.457	0.491
HTbZIP24	0.4487	0.2015	0.3371	0.2734	0.196	-0.029	0.4	55.86	0.373	0.461	0.575	0.402	0.489
HTbZIP25	0.4375	0.1821	0.3324	0.3187	0.193	-0.11	0.369	54.66	0.387	0.444	0.503	0.422	0.463
HTbZIP26	0.2755	0.398	0.3524	0.2525	0.234	0.08	0.484	57.45	0.5	0.486	0.496	0.425	0.460
HTbZIP27	0.3644	0.2331	0.2957	0.3829	0.212	0.009	0.415	56.3	0.476	0.471	0.560	0.352	0.456
HTbZIP28	0.4286	0.125	0.3209	0.3665	0.131	-0.167	0.329	49.04	0.391	0.456	0.504	0.440	0.472
HTbZIP29	0.4686	0.1698	0.3168	0.2667	0.204	-0.069	0.396	51.19	0.355	0.486	0.559	0.515	0.537
HTbZIP30	0.3769	0.3154	0.2683	0.3621	0.158	-0.048	0.388	48.79	0.503	0.462	0.509	0.349	0.429
HTbZIP31	0.2593	0.3333	0.3372	0.3718	0.136	-0.072	0.368	61	0.528	0.44	0.425	0.336	0.381
HTbZIP32	0.4212	0.209	0.3291	0.3069	0.186	-0.083	0.383	54.49	0.403	0.465	0.546	0.417	0.482
HTbZIP33	0.2268	0.3299	0.4151	0.3333	0.187	0.014	0.427	53.18	0.496	0.457	0.486	0.370	0.428
HTbZIP34	0.3462	0.3846	0.373	0.2174	0.181	-0.055	0.389	61	0.449	0.444	0.514	0.343	0.429
HTbZIP35	0.4035	0.1527	0.3575	0.3324	0.153	-0.097	0.377	52.69	0.384	0.465	0.544	0.445	0.494
HTbZIP36	0.3672	0.2734	0.2832	0.3796	0.201	0.029	0.452	52.1	0.49	0.458	0.468	0.379	0.423
HTbZIP37	0.3829	0.1971	0.3352	0.3304	0.147	-0.149	0.333	58.41	0.417	0.461	0.518	0.425	0.471
HTbZIP38	0.4266	0.2203	0.3451	0.2649	0.186	-0.122	0.347	55.22	0.379	0.455	0.560	0.400	0.480
HTbZIP39	0.289	0.327	0.2846	0.3655	0.177	-0.021	0.414	61	0.542	0.527	0.576	0.446	0.511
HTbZIP40	0.348	0.1145	0.1928	0.5579	0.156	-0.157	0.323	45.59	0.559	0.531	0.568	0.442	0.505

HTbZIP41	0.2604	0.3125	0.53	0.2022	0.153	-0.105	0.365	53.38	0.381	0.384	0.384	0.353	0.368
HTbZIP42	0.3273	0.1909	0.3209	0.3615	0.156	-0.017	0.415	52.16	0.463	0.501	0.540	0.480	0.510
HTbZIP43	0.4309	0.1989	0.3432	0.2429	0.215	-0.026	0.413	56.98	0.358	0.484	0.552	0.515	0.534
HTbZIP44	0.4408	0.2163	0.3491	0.2439	0.168	-0.168	0.34	54.9	0.361	0.434	0.473	0.440	0.456
HTbZIP45	0.3829	0.2176	0.3458	0.2955	0.165	-0.121	0.359	58.98	0.407	0.482	0.582	0.429	0.505
HTbZIP46	0.3618	0.3158	0.3958	0.2388	0.176	-0.018	0.417	52.91	0.417	0.411	0.441	0.347	0.394
HTbZIP47	0.3724	0.2296	0.3066	0.3399	0.158	-0.105	0.361	56.75	0.452	0.476	0.513	0.438	0.475
HTbZIP48	0.3579	0.2787	0.39	0.1917	0.178	-0.079	0.377	56.08	0.381	0.466	0.540	0.454	0.497
HTbZIP49	0.3684	0.2216	0.3288	0.3058	0.212	-0.029	0.417	58.01	0.428	0.499	0.557	0.490	0.524
HTbZIP50	0.3993	0.1269	0.2238	0.4478	0.172	-0.098	0.362	46.47	0.477	0.523	0.572	0.490	0.531
HTbZIP51	0.4174	0.1589	0.3628	0.2704	0.173	-0.053	0.397	50.44	0.351	0.462	0.510	0.490	0.500
HTbZIP52	0.5254	0.1186	0.3028	0.3762	0.149	-0.183	0.327	40.42	0.354	0.379	0.399	0.360	0.379
HTbZIP53	0.4097	0.2149	0.2829	0.3618	0.174	-0.089	0.378	58.53	0.448	0.467	0.504	0.430	0.467
HTbZIP54	0.4138	0.2365	0.2787	0.3657	0.199	-0.091	0.377	53.26	0.453	0.47	0.518	0.427	0.472
HTbZIP55	0.3247	0.2511	0.3291	0.3584	0.181	-0.115	0.357	55.83	0.478	0.498	0.552	0.438	0.495
HTbZIP56	0.4206	0.215	0.2566	0.3738	0.21	-0.046	0.387	58.52	0.46	0.479	0.559	0.386	0.472
HTbZIP57	0.3218	0.3333	0.359	0.2778	0.176	-0.007	0.419	53.2	0.467	0.43	0.414	0.385	0.400
HTbZIP58	0.4046	0.2366	0.3833	0.2613	0.17	-0.041	0.39	45.07	0.377	0.411	0.473	0.364	0.418
HTbZIP59	0.4231	0.2385	0.3644	0.2685	0.164	-0.059	0.38	49.09	0.38	0.415	0.479	0.364	0.421
HTbZIP60	0.3945	0.1697	0.3263	0.3406	0.169	-0.054	0.388	53.31	0.414	0.468	0.519	0.437	0.478
HTbZIP61	0.3702	0.2813	0.4005	0.2073	0.192	-0.08	0.382	51.89	0.382	0.462	0.556	0.424	0.490
HTbZIP62	0.4164	0.2164	0.3651	0.2707	0.201	-0.084	0.38	56.12	0.378	0.463	0.528	0.466	0.497
HTbZIP63	0.3023	0.2724	0.3849	0.3039	0.16	-0.082	0.384	58.81	0.451	0.486	0.557	0.424	0.490

Table S3 Predicted miRNA target distribution for *bZIP* gens in jasmine

miRNA	Target_Gene name	Target_start	Target_end	Expectation
ath-miR5658	SJbZIP62	115	135	2.5
	DJbZIP61	112	132	
	HTbZIP61	202	222	
ath-miR159b-5p	SJbZIP18	109	129	3
	DJbZIP19			
	HTbZIP18			
ath-miR414	SJbZIP50	726	746	
	DJbZIP49			
	HTbZIP49			
ath-miR778	SJbZIP39	63	83	
	DJbZIP38			
	HTbZIP38			
ath-miR865-3p	SJbZIP19	75	95	
	DJbZIP20			
	HTbZIP19			
ath-miR394a	SJbZIP38	594	613	3.5
	DJbZIP37			
	HTbZIP37			

ath-miR394b-5p	SJbZIP38			
	DJbZIP37			
	HTbZIP37			
ath-miR397b	SJbZIP54	1176	1196	
	DJbZIP53			
	HTbZIP53			
ath-miR414	HTbZIP12	414	434	
ath-miR5024-3p	SJbZIP21	557	577	
	DJbZIP22			
	HTbZIP21			
	SJbZIP63	1328	1350	
	DJbZIP62			
	HTbZIP62	1271	1293	
ath-miR5654-5p	SJbZIP03	962	981	
	DJbZIP04	1025	1044	
	HTbZIP04			
ath-miR5658	SJbZIP52	908	928	
	DJbZIP51			
	HTbZIP51			
ath-miR779.2	SJbZIP44	1107	1127	
	DJbZIP43			
	HTbZIP43	1305	1325	
ath-miR833b	SJbZIP24	766	786	
	DJbZIP31			
	HTbZIP24	727	747	
ath-miR837-5p	SJbZIP10	1143	1163	
	DJbZIP11	1146	1166	
	HTbZIP11	1140	1160	
ath-miR840-3p	SJbZIP06	300	320	
	DJbZIP07			
	HTbZIP07			
ath-miR157a-3p/b-3p	SJbZIP48	583	603	4
	DJbZIP47			
	HTbZIP47			
ath-miR157c-3p	SJbZIP18	327	347	
	DJbZIP19			
	HTbZIP18			
ath-miR158a-5p	SJbZIP18	400	420	
	DJbZIP19			
	HTbZIP18			
ath-miR169a-3p	SJbZIP38	208	227	
	DJbZIP37			
	HTbZIP37			

ath-miR169b-3p	SJbZIP13<br>DJbZIP14<br>HTbZIP14	825	846
ath-miR393a-3p	SJbZIP09<br>DJbZIP10<br>HTbZIP10	2199	2219
ath-miR393b-3p	SJbZIP64	3	23
	DJbZIP63	27	47
	HTbZIP63	3	23
ath-miR397b	SJbZIP18<br>DJbZIP19<br>HTbZIP18	95	115
ath-miR414	SJbZIP21<br>DJbZIP22<br>HTbZIP21	128	148
ath-miR417	SJbZIP20	632	652
	SJbZIP43	27	47
	DJbZIP21	632	652
	DJbZIP42	27	47
	HTbZIP20	632	652
	HTbZIP42	27	47
ath-miR4239	SJbZIP03	342	362
	DJbZIP04	405	425
	HTbZIP04		425
ath-miR5012	SJbZIP54<br>DJbZIP53<br>HTbZIP53	185	205
ath-miR5018	SJbZIP62	907	930
	DJbZIP61	904	927
	HTbZIP61	1000	1023
ath-miR5643a/b	SJbZIP19<br>DJbZIP20<br>HTbZIP19	260	280
ath-miR5645a/b/c/d/e/f	SJbZIP04<br>HTbZIP05	178	197
ath-miR5998a	SJbZIP63<br>DJbZIP62	1054	1074
	HTbZIP62	997	1017
ath-miR5998b	SJbZIP63<br>DJbZIP62	1054	1074
	HTbZIP62	997	1017
ath-miR776	SJbZIP38<br>DJbZIP37<br>HTbZIP37	153	173

ath-miR781a/b	SJbZIP51<br>DJbZIP50	119	139
	HTbZIP50	233	253
ath-miR8173	SJbZIP50<br>DJbZIP49<br>HTbZIP49	439	459
	SJbZIP22<br>DJbZIP23<br>HTbZIP22	261	281
ath-miR832-3p	SJbZIP10	771	791
	DJbZIP11	774	794
	HTbZIP11	768	788
ath-miR834	DJbZIP48	92	112
ath-miR837-5p	SJbZIP57<br>DJbZIP58<br>HTbZIP56	177	197
ath-miR861-5p	SJbZIP54<br>HTbZIP53	235	255
ath-miR868-3p	SJbZIP45<br>DJbZIP44<br>HTbZIP44	198	218

Table S4 Statistical of abscisic acid response elements of three jasmine *bZIP* genes

Gene name	Elements	Sequence	Type
SJbZIP01	ABRE	CACGTG	abscisic acid responsiveness
SJbZIP01	ABRE	ACGTG	abscisic acid responsiveness
SJbZIP02	ABRE	ACGTG	abscisic acid responsiveness
SJbZIP05	ABRE	ACGTG	abscisic acid responsiveness
SJbZIP05	ABRE	ACGTG	abscisic acid responsiveness
SJbZIP07	ABRE	ACGTG	abscisic acid responsiveness
SJbZIP07	ABRE	ACGTG	abscisic acid responsiveness
SJbZIP12	ABRE	ACGTG	abscisic acid responsiveness
SJbZIP13	ABRE	ACGTG	abscisic acid responsiveness
SJbZIP13	ABRE	ACGTG	abscisic acid responsiveness
SJbZIP14	ABRE	ACGTG	abscisic acid responsiveness
SJbZIP16	ABRE	ACGTG	abscisic acid responsiveness
SJbZIP17	ABRE	ACGTG	abscisic acid responsiveness
SJbZIP17	ABRE	ACGTG	abscisic acid responsiveness
SJbZIP18	ABRE	ACGTG	abscisic acid responsiveness
SJbZIP18	ABRE	ACGTG	abscisic acid responsiveness
SJbZIP21	ABRE	ACGTG	abscisic acid responsiveness
SJbZIP23	ABRE	ACGTG	abscisic acid responsiveness

SJbZIP25	ABRE	ACGTG	abscisic acid responsiveness
SJbZIP26	ABRE	ACGTG	abscisic acid responsiveness
SJbZIP27	ABRE	GCAACGTGTC	abscisic acid responsiveness
SJbZIP28	ABRE	GCAACGTGTC	abscisic acid responsiveness
SJbZIP30	ABRE	AACCCGG	abscisic acid responsiveness
SJbZIP31	ABRE	ACGTG	abscisic acid responsiveness
SJbZIP31	ABRE	CACGTG	abscisic acid responsiveness
SJbZIP31	ABRE	ACGTG	abscisic acid responsiveness
SJbZIP33	ABRE	ACGTG	abscisic acid responsiveness
SJbZIP33	ABRE	ACGTG	abscisic acid responsiveness
SJbZIP34	ABRE	ACGTG	abscisic acid responsiveness
SJbZIP34	ABRE	ACGTG	abscisic acid responsiveness
SJbZIP34	ABRE	GCAACGTGTC	abscisic acid responsiveness
SJbZIP34	ABRE	CACGTG	abscisic acid responsiveness
SJbZIP34	ABRE	ACGTG	abscisic acid responsiveness
SJbZIP35	ABRE	ACGTG	abscisic acid responsiveness
SJbZIP36	ABRE	ACGTG	abscisic acid responsiveness
SJbZIP36	ABRE	CACGTG	abscisic acid responsiveness
SJbZIP36	ABRE	ACGTG	abscisic acid responsiveness
SJbZIP37	ABRE	ACGTG	abscisic acid responsiveness
SJbZIP38	ABRE	ACGTG	abscisic acid responsiveness
SJbZIP38	ABRE	GCCGCGTGGC	abscisic acid responsiveness
SJbZIP40	ABRE	ACGTG	abscisic acid responsiveness
SJbZIP40	ABRE	CACGTG	abscisic acid responsiveness
SJbZIP40	ABRE	ACGTG	abscisic acid responsiveness
SJbZIP41	ABRE	ACGTG	abscisic acid responsiveness
SJbZIP41	ABRE	ACGTG	abscisic acid responsiveness
SJbZIP43	ABRE	ACGTG	abscisic acid responsiveness
SJbZIP44	ABRE	ACGTG	abscisic acid responsiveness
SJbZIP44	ABRE	ACGTG	abscisic acid responsiveness
SJbZIP45	ABRE	ACGTG	abscisic acid responsiveness
SJbZIP46	ABRE	CGCACGTGTC	abscisic acid responsiveness
SJbZIP46	ABRE	CACGTG	abscisic acid responsiveness
SJbZIP46	ABRE	ACGTG	abscisic acid responsiveness
SJbZIP47	ABRE	ACGTG	abscisic acid responsiveness
SJbZIP48	ABRE	CACGTG	abscisic acid responsiveness
SJbZIP48	ABRE	ACGTG	abscisic acid responsiveness
SJbZIP50	ABRE	ACGTG	abscisic acid responsiveness
SJbZIP53	ABRE	CACGTG	abscisic acid responsiveness
SJbZIP53	ABRE	ACGTG	abscisic acid responsiveness
SJbZIP54	ABRE	CACGTG	abscisic acid responsiveness
SJbZIP54	ABRE	ACGTG	abscisic acid responsiveness
SJbZIP56	ABRE	CACGTG	abscisic acid responsiveness
SJbZIP56	ABRE	ACGTG	abscisic acid responsiveness

SJbZIP57	ABRE	CACGTG	abscisic acid responsiveness
SJbZIP57	ABRE	ACGTG	abscisic acid responsiveness
SJbZIP58	ABRE	ACGTG	abscisic acid responsiveness
SJbZIP59	ABRE	ACGTG	abscisic acid responsiveness
SJbZIP59	ABRE	ACGTG	abscisic acid responsiveness
SJbZIP61	ABRE	ACGTG	abscisic acid responsiveness
SJbZIP62	ABRE	ACGTG	abscisic acid responsiveness
SJbZIP63	ABRE	ACGTG	abscisic acid responsiveness
SJbZIP63	ABRE	ACGTG	abscisic acid responsiveness
SJbZIP63	ABRE	GACACGTGGC	abscisic acid responsiveness
SJbZIP63	ABRE	CACGTG	abscisic acid responsiveness
SJbZIP63	ABRE	ACGTG	abscisic acid responsiveness
SJbZIP63	ABRE	GCAACGTGTC	abscisic acid responsiveness
SJbZIP63	ABRE	CACGTG	abscisic acid responsiveness
SJbZIP63	ABRE	ACGTG	abscisic acid responsiveness
SJbZIP63	ABRE	TACGTGTC	abscisic acid responsiveness
SJbZIP63	ABRE	ACGTG	abscisic acid responsiveness
DJbZIP01	ABRE	ACGTG	abscisic acid responsiveness
DJbZIP01	ABRE	CACGTG	abscisic acid responsiveness
DJbZIP01	ABRE	ACGTG	abscisic acid responsiveness
DJbZIP02	ABRE	ACGTG	abscisic acid responsiveness
DJbZIP03	ABRE	ACGTG	abscisic acid responsiveness
DJbZIP04	ABRE	ACGTG	abscisic acid responsiveness
DJbZIP04	ABRE	ACGTG	abscisic acid responsiveness
DJbZIP06	ABRE	ACGTG	abscisic acid responsiveness
DJbZIP07	ABRE	CACGTG	abscisic acid responsiveness
DJbZIP07	ABRE	ACGTG	abscisic acid responsiveness
DJbZIP08	ABRE	ACGTG	abscisic acid responsiveness
DJbZIP08	ABRE	ACGTG	abscisic acid responsiveness
DJbZIP13	ABRE	ACGTG	abscisic acid responsiveness
DJbZIP14	ABRE	ACGTG	abscisic acid responsiveness
DJbZIP14	ABRE	ACGTG	abscisic acid responsiveness
DJbZIP15	ABRE	ACGTG	abscisic acid responsiveness
DJbZIP17	ABRE	ACGTG	abscisic acid responsiveness
DJbZIP18	ABRE	ACGTG	abscisic acid responsiveness
DJbZIP18	ABRE	ACGTG	abscisic acid responsiveness
DJbZIP19	ABRE	ACGTG	abscisic acid responsiveness
DJbZIP19	ABRE	ACGTG	abscisic acid responsiveness
DJbZIP22	ABRE	ACGTG	abscisic acid responsiveness
DJbZIP24	ABRE	ACGTG	abscisic acid responsiveness
DJbZIP25	ABRE	CACGTG	abscisic acid responsiveness
DJbZIP25	ABRE	ACGTG	abscisic acid responsiveness
DJbZIP25	ABRE	ACGTG	abscisic acid responsiveness
DJbZIP26	ABRE	ACGTG	abscisic acid responsiveness

DJbZIP28	ABRE	GCAACGTGTC	abscisic acid responsiveness
DJbZIP29	ABRE	ACGTG	abscisic acid responsiveness
DJbZIP30	ABRE	ACGTG	abscisic acid responsiveness
DJbZIP33	ABRE	ACGTG	abscisic acid responsiveness
DJbZIP33	ABRE	ACGTG	abscisic acid responsiveness
DJbZIP33	ABRE	GCAACGTGTC	abscisic acid responsiveness
DJbZIP33	ABRE	CACGTG	abscisic acid responsiveness
DJbZIP33	ABRE	ACGTG	abscisic acid responsiveness
DJbZIP34	ABRE	ACGTG	abscisic acid responsiveness
DJbZIP35	ABRE	ACGTG	abscisic acid responsiveness
DJbZIP35	ABRE	CACGTG	abscisic acid responsiveness
DJbZIP35	ABRE	ACGTG	abscisic acid responsiveness
DJbZIP36	ABRE	ACGTG	abscisic acid responsiveness
DJbZIP37	ABRE	GCCGCGTGGC	abscisic acid responsiveness
DJbZIP38	ABRE	ACGTG	abscisic acid responsiveness
DJbZIP39	ABRE	ACGTG	abscisic acid responsiveness
DJbZIP39	ABRE	CACGTG	abscisic acid responsiveness
DJbZIP39	ABRE	ACGTG	abscisic acid responsiveness
DJbZIP39	ABRE	ACGTG	abscisic acid responsiveness
DJbZIP40	ABRE	ACGTG	abscisic acid responsiveness
DJbZIP40	ABRE	ACGTG	abscisic acid responsiveness
DJbZIP42	ABRE	ACGTG	abscisic acid responsiveness
DJbZIP43	ABRE	ACGTG	abscisic acid responsiveness
DJbZIP43	ABRE	ACGTG	abscisic acid responsiveness
DJbZIP44	ABRE	ACGTG	abscisic acid responsiveness
DJbZIP45	ABRE	CGCACGTGTC	abscisic acid responsiveness
DJbZIP45	ABRE	CACGTG	abscisic acid responsiveness
DJbZIP45	ABRE	ACGTG	abscisic acid responsiveness
DJbZIP46	ABRE	ACGTG	abscisic acid responsiveness
DJbZIP47	ABRE	CACGTG	abscisic acid responsiveness
DJbZIP47	ABRE	ACGTG	abscisic acid responsiveness
DJbZIP49	ABRE	ACGTG	abscisic acid responsiveness
DJbZIP50	ABRE	TACGGTC	abscisic acid responsiveness
DJbZIP52	ABRE	CACGTG	abscisic acid responsiveness
DJbZIP52	ABRE	ACGTG	abscisic acid responsiveness
DJbZIP53	ABRE	CACGTG	abscisic acid responsiveness
DJbZIP53	ABRE	ACGTG	abscisic acid responsiveness
DJbZIP55	ABRE	CACGTG	abscisic acid responsiveness
DJbZIP55	ABRE	ACGTG	abscisic acid responsiveness
DJbZIP56	ABRE	ACGTG	abscisic acid responsiveness
DJbZIP56	ABRE	ACGTG	abscisic acid responsiveness
DJbZIP57	ABRE	ACGTG	abscisic acid responsiveness
DJbZIP58	ABRE	CACGTG	abscisic acid responsiveness
DJbZIP58	ABRE	ACGTG	abscisic acid responsiveness

DJbZIP60	ABRE	ACGTG	abscisic acid responsiveness
DJbZIP62	ABRE	ACGTG	abscisic acid responsiveness
DJbZIP62	ABRE	ACGTG	abscisic acid responsiveness
DJbZIP62	ABRE	GACACGTGGC	abscisic acid responsiveness
DJbZIP62	ABRE	CACGTG	abscisic acid responsiveness
DJbZIP62	ABRE	ACGTG	abscisic acid responsiveness
DJbZIP62	ABRE	TACGTGTC	abscisic acid responsiveness
DJbZIP62	ABRE	ACGTG	abscisic acid responsiveness
HTbZIP39	ABRE	ACGTG	abscisic acid responsiveness
HTbZIP18	ABRE	ACGTG	abscisic acid responsiveness
HTbZIP18	ABRE	ACGTG	abscisic acid responsiveness
HTbZIP38	ABRE	ACGTG	abscisic acid responsiveness
HTbZIP08	ABRE	ACGTG	abscisic acid responsiveness
HTbZIP07	ABRE	CACGTG	abscisic acid responsiveness
HTbZIP07	ABRE	ACGTG	abscisic acid responsiveness
HTbZIP03	ABRE	ACGTG	abscisic acid responsiveness
HTbZIP43	ABRE	CACGTG	abscisic acid responsiveness
HTbZIP43	ABRE	ACGTG	abscisic acid responsiveness
HTbZIP13	ABRE	ACGTG	abscisic acid responsiveness
HTbZIP29	ABRE	ACGTG	abscisic acid responsiveness
HTbZIP36	ABRE	ACGTG	abscisic acid responsiveness
HTbZIP56	ABRE	CACGTG	abscisic acid responsiveness
HTbZIP56	ABRE	ACGTG	abscisic acid responsiveness
HTbZIP08	ABRE	ACGTG	abscisic acid responsiveness
HTbZIP15	ABRE	ACGTG	abscisic acid responsiveness
HTbZIP14	ABRE	CACGTG	abscisic acid responsiveness
HTbZIP14	ABRE	ACGTG	abscisic acid responsiveness
HTbZIP62	ABRE	ACGTG	abscisic acid responsiveness
HTbZIP04	ABRE	ACGTG	abscisic acid responsiveness
HTbZIP32	ABRE	ACGTG	abscisic acid responsiveness
HTbZIP44	ABRE	ACGTG	abscisic acid responsiveness
HTbZIP06	ABRE	ACGTG	abscisic acid responsiveness
HTbZIP01	ABRE	CACGTG	abscisic acid responsiveness
HTbZIP01	ABRE	ACGTG	abscisic acid responsiveness
HTbZIP52	ABRE	CACGTG	abscisic acid responsiveness
HTbZIP35	ABRE	ACGTG	abscisic acid responsiveness
HTbZIP52	ABRE	ACGTG	abscisic acid responsiveness
HTbZIP47	ABRE	CACGTG	abscisic acid responsiveness
HTbZIP32	ABRE	ACGTG	abscisic acid responsiveness
HTbZIP47	ABRE	ACGTG	abscisic acid responsiveness
HTbZIP02	ABRE	ACGTG	abscisic acid responsiveness
HTbZIP08	ABRE	ACGTG	abscisic acid responsiveness
HTbZIP37	ABRE	GCCGCGTGGC	abscisic acid responsiveness
HTbZIP53	ABRE	CACGTG	abscisic acid responsiveness

HTbZIP53	ABRE	ACGTG	abscisic acid responsiveness
HTbZIP26	ABRE	ACGTG	abscisic acid responsiveness
HTbZIP33	ABRE	ACGTG	abscisic acid responsiveness
HTbZIP17	ABRE	ACGTG	abscisic acid responsiveness
HTbZIP33	ABRE	ACGTG	abscisic acid responsiveness
HTbZIP39	ABRE	CACGTG	abscisic acid responsiveness
HTbZIP39	ABRE	ACGTG	abscisic acid responsiveness
HTbZIP30	ABRE	CACGTG	abscisic acid responsiveness
HTbZIP30	ABRE	ACGTG	abscisic acid responsiveness
HTbZIP30	ABRE	ACGTG	abscisic acid responsiveness
HTbZIP14	ABRE	ACGTG	abscisic acid responsiveness
HTbZIP23	ABRE	ACGTG	abscisic acid responsiveness
HTbZIP59	ABRE	ACGTG	abscisic acid responsiveness
HTbZIP45	ABRE	CGCACGTGTC	abscisic acid responsiveness
HTbZIP40	ABRE	ACGTG	abscisic acid responsiveness
HTbZIP45	ABRE	CACGTG	abscisic acid responsiveness
HTbZIP45	ABRE	ACGTG	abscisic acid responsiveness
HTbZIP55	ABRE	CACGTG	abscisic acid responsiveness
HTbZIP55	ABRE	ACGTG	abscisic acid responsiveness
HTbZIP49	ABRE	CACGTG	abscisic acid responsiveness
HTbZIP49	ABRE	ACGTG	abscisic acid responsiveness
HTbZIP13	ABRE	ACGTG	abscisic acid responsiveness
HTbZIP42	ABRE	ACGTG	abscisic acid responsiveness
HTbZIP35	ABRE	CACGTG	abscisic acid responsiveness
HTbZIP35	ABRE	ACGTG	abscisic acid responsiveness
HTbZIP21	ABRE	ACGTG	abscisic acid responsiveness
HTbZIP04	ABRE	ACGTG	abscisic acid responsiveness
HTbZIP40	ABRE	ACGTG	abscisic acid responsiveness
HTbZIP62	ABRE	ACGTG	abscisic acid responsiveness
HTbZIP27	ABRE	GCAACGTGTC	abscisic acid responsiveness
HTbZIP62	ABRE	GACACGTGGC	abscisic acid responsiveness
HTbZIP62	ABRE	CACGTG	abscisic acid responsiveness
HTbZIP62	ABRE	ACGTG	abscisic acid responsiveness
HTbZIP34	ABRE	ACGTG	abscisic acid responsiveness
HTbZIP33	ABRE	GCAACGTGTC	abscisic acid responsiveness
HTbZIP33	ABRE	CACGTG	abscisic acid responsiveness
HTbZIP33	ABRE	ACGTG	abscisic acid responsiveness
HTbZIP62	ABRE	TACGTGTC	abscisic acid responsiveness
HTbZIP62	ABRE	ACGTG	abscisic acid responsiveness
HTbZIP25	ABRE	ACGTG	abscisic acid responsiveness
HTbZIP57	ABRE	ACGTG	abscisic acid responsiveness
HTbZIP58	ABRE	ACGTG	abscisic acid responsiveness
HTbZIP59	ABRE	ACGTG	abscisic acid responsiveness

Table S5 RT-PCR primer design

Gene Name	Sequence
SJbZIP05F	TACAGCAGTCGTCTCACCAAGC
SJbZIP05R	CAGGTCCCGAAGATCCAGTAGC
SJbZIP13F	CCAAGAATGTAGGAGGTCATCAATAGG
SJbZIP13R	GTTATGATCCGATGGCGAAGAAGG
SJbZIP28F	TGACAGTGAGAAAGGAAGCAAGTAAC
SJbZIP28R	GCTAGTTGTGAAGTGGACCATCTTAG
SJbZIP37F	GAATGTGGACGCTGAGAACTCTG
SJbZIP37R	GGCGGCGGTGTTGGAATTG
SJbZIP57F	GTTGTCTTCCTCTACTGGTTCTATGTG
SJbZIP57R	TTCGGCTGATTGGTTGATTTGGG
SJbZIP62F	GTGAGAAGCCTAAGAGAACCAATCC
SJbZIP62R	GAGTAAGAGATGAGTGGTAGAAGAAGAC
DJbZIP06F	TACAGCAGTCGTCTCACCAAGC
DJbZIP06R	CAGGTCCCGAAGATCCAGTAGC
DJbZIP14F	CCAAGAATGTAGGAGGTCATCAATAGG
DJbZIP14R	GTTATGATCCGATGGCGAAGAAGG
DJbZIP28F	TGACAGTGAGAAAGGAAGCAAGTAAC
DJbZIP28R	GCTAGTTGTGAAGTGGACCATCTTAG
DJbZIP36F	GAATGTGGACGCTGAGAACTCTG
DJbZIP36R	GGCGGCGGTGTTGGAATTG
DJbZIP58F	GTTGTCTTCCTCTACTGGTTCTATGTG
DJbZIP58R	TTCGGCTGATTGGTTGATTTGGG
DJbZIP63F	GTGAGAAGCCTAAGAGAACCAATCC
DJbZIP63R	GAGTAAGAGATGAGTGGTAGAAGAAGAC
HTbZIP06F	TACAGCAGTCGTCTCACCAAGC
HTbZIP06R	CAGGTCCCGAAGATCCAGTAGC
HTbZIP14F	CCAAGAATGTAGGAGGTCATCAATAGG
HTbZIP14R	GTTATGATCCGATGGCGAAGAAGG
HTbZIP27F	TGACAGTGAGAAAGGAAGCAAGTAAC
HTbZIP27R	GCTAGTTGTGAAGTGGACCATCTTAG
HTbZIP36F	GAATGTGGACGCTGAGAACTCTG
HTbZIP36R	GGCGGCGGTGTTGGAATTG
HTbZIP56F	GTTGTCTTCCTCTACTGGTTCTATGTG
HTbZIP56R	TTCGGCTGATTGGTTGATTTGGG
HTbZIP62F	GTGAGAAGCCTAAGAGAACCAATCC
HTbZIP62R	GAGTAAGAGATGAGTGGTAGAAGAAGAC
JsActin*F*	TCTCTATGGTAACATTGTCCTG
JsActin*R*	ATCCAGACACTGTACTTCCTCT
